# Synthesis and Antimicrobial Properties of Highly Cross-Linked pH-Sensitive Hydrogels through Gamma Radiation

**DOI:** 10.3390/polym13142223

**Published:** 2021-07-06

**Authors:** Moises Bustamante-Torres, Victor H. Pino-Ramos, David Romero-Fierro, Sandra P. Hidalgo-Bonilla, Héctor Magaña, Emilio Bucio

**Affiliations:** 1 Departamento de Biología, Escuela de Ciencias Biológicas e Ingeniería, Universidad de Investigación de Tecnología Experimental Yachay, Hacienda San José s/n y Proyecto Yachay (Ciudad del Conocimiento Yachay), Urcuquí 100650, Ecuador; 2 Departamento de Química de Radiaciones y Radioquímica, Instituto de Ciencias Nucleares, Universidad Nacional Autónoma de México, Circuito Exterior, Ciudad Universitaria, Ciudad de México 04510, Mexico; vickor_ari@hotmail.es; 3 Departamento de Química, Escuela de Ciencias Química e Ingeniería, Universidad de Investigación de Tecnología Experimental Yachay, Hacienda San José s/n y Proyecto Yachay (Ciudad del Conocimiento Yachay), Urcuquí 100650, Ecuador; david.romero@yachaytech.edu.ec (D.R.-F.); sahidalgo@yachaytech.edu.ec (S.P.H.-B.); 4 Facultad de Ciencias Químicas e Ingeniería, Universidad Autónoma de Baja California, Calzada Universidad 14418, Parque Industrial Internacional Tijuana, Tijuana 22390, Mexico; hector.magana@uabc.edu.mx

**Keywords:** hydrogel, smart polymers, drug delivery, pH-sensitive polymers, gamma radiation, cross-linking, synthesis, characterization

## Abstract

The design of new polymeric systems for antimicrobial drug release focused on medical/surgical procedures is of great interest in the biomedical area due to the high prevalence of bacterial infections in patients with wounds or burns. For this reason, in this work, we present a new design of pH-sensitive hydrogels copolymerized by a graft polymerization method (gamma rays), intended for localized prophylactic release of ciprofloxacin and silver nanoparticles (AgNPs) for potential topical bacterial infections. The synthesized hydrogels were copolymerized from acrylic acid (AAc) and agar. Cross-linked hydrogel film formation depended on monomer concentrations and the degree of radiation used (Cobalt-60). The obtained hydrogel films were characterized by attenuated total reflectance Fourier-transform infrared spectroscopy (ATR-FTIR), thermogravimetric analysis (TGA), differential scanning calorimetry (DSC), and mechanical testing. The swelling of the hydrogels was evidenced by the influence of their pH-sensitiveness. The hydrogel was loaded with antimicrobial agents (AgNPs or ciprofloxacin), and their related activity was evaluated. Finally, the antimicrobial activity of biocidal-loaded hydrogel was tested against *Escherichia coli* (*E. coli*) and *methicillin-resistant Staphylococcus aureus* (*MRSA*) on in vitro conditions.

## 1. Introduction

Smart polymers or stimulus-responsive polymers are those with the ability to change their physical conformation by variations in the external stimuli that surround them, such as pH, temperature, ionic strength and electrochemical fields [1]. Some of these sensitive polymers have been targeted for the development of hydrogels [2]. A hydrogel is a tridimensional polymeric structure with swollen and collapse properties, flexibility, biodegradability, biocompatibility and softness [3]. These characteristics have made hydrogels suitable for biomedical applications, such as drug delivery systems, medical devices, sensors and contact lenses, and have attracted a great deal of attention [4].

Some of the main applications of hydrogels in pharmaceuticals and medical devices are topical patches to prevent inflammatory and infectious processes caused by wounds or burns. For this reason, contacting hydrogels with analgesic, antimicrobial or anti-inflammatory drugs is of great interest in healthcare settings. Some of the drugs that have been previously used in this system are diclofenac [5], salicylic acid [6], dexamethasone [7], and gentamicin [8]. One of the ways to heal the wound is within a moist physiological environment using active dressings. A moist environment prevents cell desiccation, stimulates angiogenesis and collagen synthesis, thus enabling cell migration and intercellular communication [9]. All of this translates into less pain, thermal insulation, autolytic debridement, speedy healing, and better scarring [10]. An ideal dressing should maintain a moist physiological environment; it needs to be an insulating and protective barrier; it should allow gas exchange, adequate blood circulation, and remove secretions. Hydrogel dressings are considered for this healing method [11] since they act as an effective barrier to prevent environmental bacteria from infecting the wound [12]. Conventional methods usually involve water-based treatments, fluids to prevent dehydration, dressings, and drugs to treat infections or, in worst cases, surgical interventions. Nevertheless, hydrogels can absorb water and any substance diluted in them. Moreover, it is possible to insert active components allowing better and faster healing.

The pH-sensitive hydrogels are those that swell-collapse (depends on the ionizable group that contains it) in response to changes in the pH of their environment; this causes a change in the degree of ionization of the polymer, which in turn causes a readjustment in the hydrophilic/hydrophobic interactions on the polymer chains [13].

Bacterial infections are caused by either gram-positive or gram-negative microorganisms, leading to widespread mortality and the overload of the global medical system [14,15]. A drug-loaded stimulus-sensitive hydrogel might have the ability to release a drug to be in direct contact with wounds or burns in a specific area, enhancing the availability of the drug [16,17,18].

The swell–collapse behavior depends on the functional groups present in the hydrogel structure, such as carboxylic acids/polyanions (R–COOH) or amines/polycations (R-NH2) [19]. The pKa of the ionizable groups present on the polymeric chains is very important since, depending on the pH, it will present interchain electrostatic repulsion, generating a swelling effect in the hydrogel [20,21,22,23,24]. Some examples of polymers that present this behavior are poly(methacrylic acid) (PMAA) and poly(acrylic acid) (PAAc). The latter also presents mucoadhesive/bioadhesive properties. For that reason, it has been of great interest for developing drug delivery systems.

Agar is another polymer that has been used for hydrogel productions in the medical, cosmetic and food fields. This biopolymer is a collective term used to describe a mixture of colloidal polysaccharides composed of D- and L-galactose (agarose) [25]. One of the essential characteristics of agar is that it can form a reversible gel by cooling it in a hot water solution even at low concentrations due to its ability to create hydrogen bonds [26,27]. This property makes it an excellent candidate for the synthesis of hydrogels.

The grafting-radiation method is a physical technique that exhibits advantages over chemical methods since it does not need chemical initiators to induce cross-linking reactions. Besides, the radiation energy can be applied in aerobic conditions at room temperature. When vinylic monomers in solution are exposed to an energy source, radicals boost a polymeric reaction. This process is traditionally carried out by accelerated electrons (E-Beam) or gamma radiation (60-Cobalt).

This study aimed to prepare Agar/AAc hydrogels by free radical polymerization using γ-rays as a cross-linking agent. The hydrogels presented different degrees of cross-linking as a function of monomers content and the applied radiation dose. The hydrogel films were characterized by ATR-FITR, TGA, DSC, and mechanical testing (Young’s modulus). The synthesized hydrogels exhibited excellent water uptake capacity without getting deformed; besides, they presented a critical pH. Physical interactions were used to load the silver nanoparticles (NPs) and ciprofloxacin into the hydrogel. The antimicrobial activity was evaluated in *E. coli* and *MRSA* for potential applications in medical and surgical procedures.

## 2. Materials and Methods

### 2.1. Materials

Agar (used as received), acrylic acid (purified by distillation before use), sodium citrate, silver nitrate, and sodium hydroxide were purchased from Sigma-Aldrich (St. Louis, MO, USA). Citric acid, boric acid, and trisodium phosphate dodecahydrate were acquired from J.T. Baker (CDMX, Mexico). *E. coli* (ATCC 31165) and *S. aureus* (ATCC 29213) were kindly provided by the Microbiology Department of the Chemistry School of the National Autonomous University of Mexico. Bidistilled water was used in all the experiments.

### 2.2. Preparation of Hydrogels

The synthesis of poly(Agar-*co*-AAc) hydrogels was performed by dissolving 0.8 g of agar in 50 mL of deionized water under constant stirring, close to the boiling point until a homogeneous solution was obtained. Then, while constantly stirring, predetermined vol-umes of AAc aqueous solutions were added to 5 mL of freshly prepared agar solution drop by drop. The concentrations (*v*/*v*%) of agar and AAc monomer are described in Table 1. Later, the final solution was immediately transferred to a petri dish and cooled at room temperature. This process was repeated for different AAc concentrations to obtain different hydrogels. Then, the petri dish was irradiated with a pre-established irradiation dose to induce the cross-linking process; the dose intensity used was 7.1 kGy/h. After the irradiation process, the hydrogels were washed in distilled water under constant stirring for one hour (3 times) to remove the formed by-products. Finally, the samples were dried by lyophilization in a Freeze Dry System/model Freezone 4.5 LABCONCO and stored until use.

### 2.3. Synthesis of Silver Nanoparticles

The synthesis of the metallic nanoparticles was mainly based on the method previously reported by Lee and Meisel [28]. First, 45 mg of silver nitrate (AgNO_3_) was dissolved in 250 mL of distilled water with constant stirring. Then, drops of NaOH 0.1 M solution were added to adjust the solution to pH 8. After this, 5 mL of sodium citrate solution (C_6_H_5_Na_3_O_7_) 1% was added drop by drop; the mixture was kept close to the water boiling point for one hour under constant stirring. The solution acquired a brown color: then, it was cooled at room temperature. Subsequently, 10 mL of the final solution was taken and adjusted to 100 mL; the absorbance of the final solution was measured in a UV-vis spectrophotometer at 416 nm.

### 2.4. Characterization

#### 2.4.1. ATR-FTIR Analysis

Agar, AAc, and poly(Agar-*co*-AAc) hydrogel (10–15 mg) were characterized by ATR-FTIR, using 16 scans from 4000 to 650 cm^−1^. This characterization was carried out using a spectrometer, Spectrum 100 (Perkin-Elmer Cetus Instruments, Norwalk, CT, USA). The frequency of each band obtained in the spectra was assigned with the software.

#### 2.4.2. Thermal Analysis

The thermal properties of Agar, AAc, and poly (Agar-*co*-AAc) hydrogel were evaluated by Differential Scanning Calorimetry (DSC) and Thermogravimetric Analysis (TGA). In reference to DSC experiments, 5–10 mg of each sample was encapsulated in a hermetically sealed aluminum pan; then, it was placed into the oven of DSC Q|00 (TA Instruments, New Castle, DE, USA) equipment. The first heating procedure was carried out at 20 to 100 °C to remove some residual water or moisture, while the second heating cycle was carried out at 25 to 300 °C to observe the *T*_m_ and *T*_g_ of the sample. All experiments were carried out within an inert atmosphere (nitrogen), and the heat flow rate was 5 °C/min. On the other hand, the thermogravimetric analysis was carried out in a TGA Q50 (TA Instruments, New Castle, DE, USA) under nitrogen atmosphere; for this, samples from 5 to 15 mg were analyzed using a heating ramp of 10 °C/min from 25 to 900 °C.

#### 2.4.3. Mechanical Testing

Hydrogel films of poly(Agar-*co*-AAc) were adequately cut and then subjected to tensile tests to get and compare, in this case, the Young’s modulus and deformation percentage of different samples. After that irradiation process, the samples were cut and evaluated in an autograph AGS-X-Shimadzu (Tokyo, Japan), using the following conditions: 44% moisture, the rate at 10 mm/min, and room temperature. The thickness of the analyzed samples consisted of a range of 0.90–2.20 mm. The measurements were repeated, at least, three times for each hydrogel.

#### 2.4.4. Swelling Studies

Hydrogel circles of 1 cm in diameter, previously dried, were studied to determine their water adsorption capacity. The study was performed by immersing the samples in distilled water at room temperature. The mass gain was measured gravimetrically by intervals of time from 15 min until 48 h of immersion; in each measurement, the sample was taken out from the distilled water and weighed after removing excess water with an absorbent paper. The following equation was used to calculate the swelling percentage:

Swelling (%) = ((X_H_ − X_I_)/X_I_) × 100 (1)

where X_H_ is the weight of the hydrogel swelled, while X_I_ is the weight of the dry hydrogel. When the weight of the hydrogel remains constant, the maximum swelling is determined.

#### 2.4.5. Critical pH

On the analyte, the samples were washed with distilled water to remove possible by-products and were then dried to a constant weight in an oven vacuum at 50 °C. Then, these samples were immersed in phosphate buffer solutions (PBS) of different pH. The PBS (pH from 2 to 12) was prepared by mixing an appropriate amount of boric acid (0.2 M) and citric acid (0.05 M) solution with a solution of trisodium phosphate dodecahydrate (0.1 M). Once the swelling equilibrium was reached in each buffer solution, each sample was removed from the vial. The experiment was carried out at room temperature three times.

#### 2.4.6. Load of Antimicrobial Compounds

Hydrogel samples were cut into circles (1 cm diameter) and washed in distilled water for 4 h to remove possible impurities in the hydrogels; then, they were dried until a constant weight. Subsequently, those cut samples were placed in 4 mL of AgNPs (0.0784 mg/mL) or 4 mL of ciprofloxacin (0.012 mg/mL) solution, stirred for 45 h at room temperature, and protected from light during the test. The amount of loaded ciprofloxacin and AgNPs was calculated by UV-visible spectroscopy (Analytikjena Specord 200 Plus). The absorbance of AgNPs and ciprofloxacin were measured at 416 and 260 nm, respectively. The amount loaded of each antimicrobial agent was calculated by the following equation:

Antimicrobial loaded (mg/g) = ((C_1_ − C_2_) × V)/W (2)

where C_1_ and C_2_ represent the initial and final concentrations of the drug solution, respectively, V the volume of solution used for loading, and W the weight of the sample.

#### 2.4.7. Antimicrobial Assay Using *E. coli* and *MRSA*

Hydrogel films (AAc/Agar-30/70, *v*/*v*%) loaded with AgNPs or ciprofloxacin were placed in a 12-well plate containing *E. coli* or *MRSA* bacterial solutions, adjusted with soybean trident broth (BD, Bioxon, Monterrey, Mexico) to 0.5 McFarland. Subsequently, the samples were incubated at 37 °C for 18 h under aerobic conditions. After the incubation time, 100 µL of each treatment medium was passed through a 96-well plate. Finally, the absorbance was measured at 600 nm using a microplate reader (Biotek, Winooski, VT, USA) to determine bacterial survival.

## 3. Results and Discussions

### 3.1. Synthesis of Hydrogels

**Figure 1:**
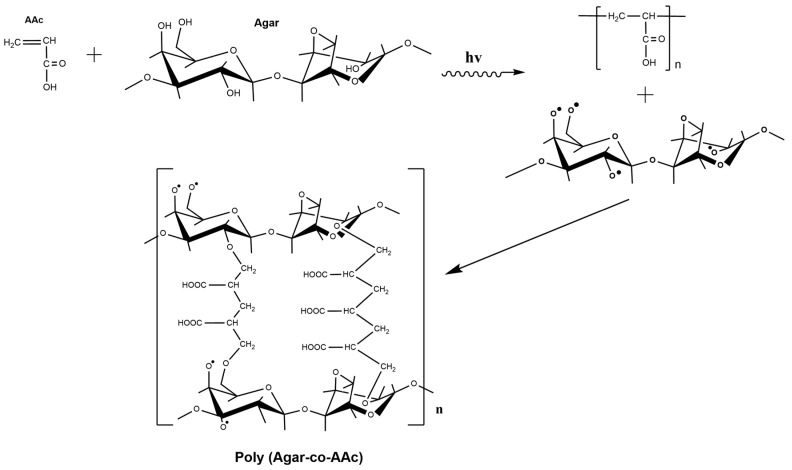
Proposed scheme between agar and acrylic acid induced by gamma rays.

Hydrogels were synthesized by a direct gamma-radiation method [29,30]. This method involves a simultaneous cross-linked polymerization of AAc and agar (one step), generating free radicals and obtaining the poly(Agar-*co*-AAc) hydrogels. Figure 1 proposes a possible and feasible structure for the poly(Agar-*co*-AAc) hydrogel.

**Figure 2:**
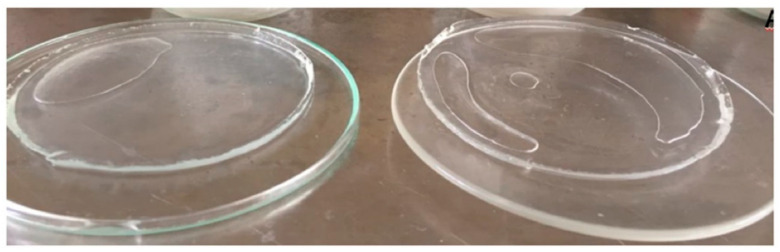
AAc-*co*-Agar hydrogels in the swollen state.

Once the agar and the AAc solution have been irradiated, the agar forms free radicals on hydroxyl groups [31] and interacts with the vinyl group present in AAc to promote polymeric cross-linking. Vinyl groups are the pathway to understanding how AAc can react with itself or even with other compounds due to its high reactivity. Figure 2 illustrates a sample of the obtained hydrogels, which were subjected to swelling, depicting insolubility characteristics. In addition, these hydrogels showed a transparent color. The uniformity was considerable because these hydrogels can increase their volume, even without losing their shape. The toughness and rigidity of the dry and swelling extent of hydrogels depend on the amount of AAc. Adhesivity is a characteristic provided by AAc, present in every sample, even those with a minimum amount of this monomer content.

### 3.2. Characterization

#### 3.2.1. ATR-FTIR Analysis

**Figure 3:**
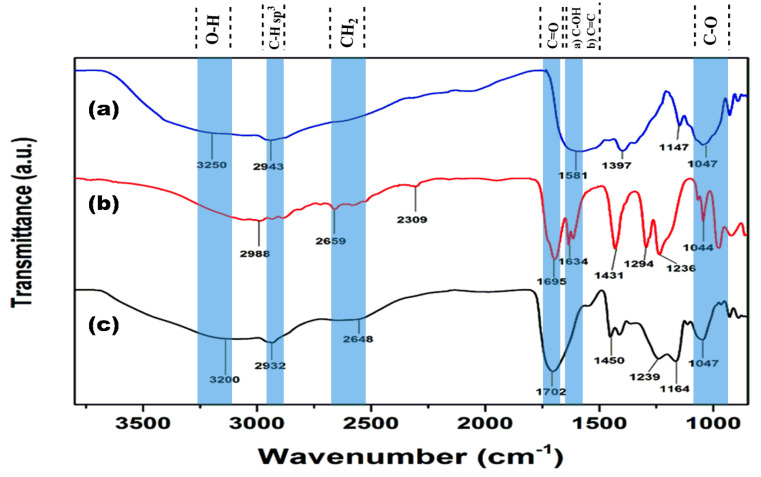
ATR-FTIR spectra of monomers and hydrogels: (**a**) Agar, (**b**) AAc, and (**c**) poly(Agar-*co*-AAc) hydrogel (AAc/Agar-30/70, *v*/*v*%).

Figure 3 illustrates the characterization by ATR-FTIR, aiming to demonstrate the cross-linking of the hydrogel. Figure 3a shows the ATR-FTIR agar spectrum broadband at 3250 cm^−1^, corresponding to the O–H stretching mode. At 2943 cm^−1^, there is a band associated with C–H sp^3^ stretching vibration. At 1581 cm^−1^, there is a band corresponding to the C–OH bending. Finally, 1047 cm^−1^ refers to the C–O bond stretching vibration. Figure 3b shows the AAc ATR-FTIR spectrum broadband that belongs to the hydroxyl group (O–H) overlapping the stretching vibrations sp^3^ of the C–H bond at 2988 cm^−1^; the band observed at 2659 cm^−1^ was associated with the CH_2_ bending mode. At 1695 cm^−1^ the signal of carbonyl (C=O) stretching vibration was observed. At 1634 cm^−1,^ a band corresponds to the C=C bending mode [32]; at 1431 cm^−1^, there is a signal corresponding to the C–H (CH_2_) bending vibrations. Two bands observed at 1294 and 1236 cm^−1^ are characteristic of the C–O bond asymmetric and symmetric stretching vibrations of the carboxylic acid. Figure 3c, corresponding to poly(Agar-*co*-AAc), presented the combination of signals of both raw monomers. The main difference is the absence of a double bond or vinyl group at 1634 cm^−1^. Thus, it can show the correct functionalization or cross-linking between AAc and agar to form the hydrogel.

#### 3.2.2. Thermal Analysis

**Figure 4:**
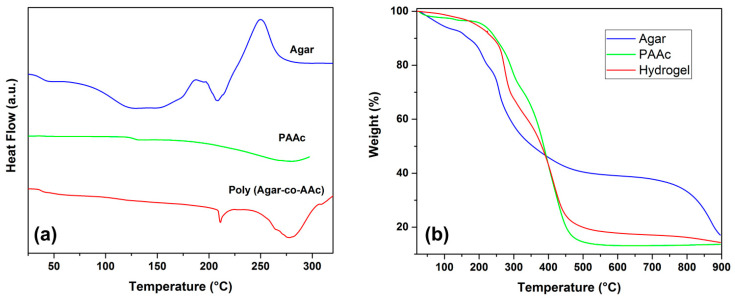
Thermal analysis: (**a**) DSC and (**b**) TGA of Agar, PAAc, and poly (Agar-*co*-AAc) (30/70, *v*/*v*%) concentration.

Figure 4a presents the thermal profile of agar, which presented a *T*_g_ at 32.9 °C, and a Tm of 207.7 °C; at around 249.5 °C, an exothermic peak was shown, which is associated with the decomposition of the material. Meanwhile, PAAc showed its *T*_g_ at 128.8 °C. Finally, the hydrogel (AAc/Agar-30/70, *v*/*v*%) presented a *T*_g_ at 37.5 °C, as well as two endothermic peaks, one at 211.0 °C and the other at 278.1 °C. The signals noticed at 37.5 °C and 278 °C are due to the presence of PAAc on the copolymer; meanwhile, the peak observed at 211 °C belongs to agar; this thermal profile displays the behavior of a new polymeric system, which demonstrates the combination of both polymers. Additionally, the hydrogel exhibited an enhancement in thermal resistance compared to agar, since no exothermic signals were observed for this sample.

Figure 4b shows the thermal stability of PAAc, agar, and poly(Agar-*co*-AAc). Agar presented three decomposition temperatures at 155.7, 257, and 868.4 °C. The first degradation starts from 50 °C and continues to above 155 °C in a 6% loss in weight due to dehydration. The prolonged weight loss of water above 100 °C is due to the strength of hydrogen bonds between hydrophilic functional groups of agar and PAAc with water from environmental moisture.

The second decomposition curve that was observed at 257 °C belongs to the degradation of agarose; it is the main component of agar, which is the reason it was the more pronounced decomposition curve. Meanwhile, the second one, at 868.4 °C, corresponds to agaropectin, the other component of the biopolymer. On the other hand, PAAc presented a decomposition step at 130 °C because of the absorbed environmental moisture, due to this polymer being highly hydrophilic and an excellent absorbent agent. The curve observed at 293.5 °C belongs to the thermal decarboxylation of PAAc; at 393 °C, the backbone degradation was observed. Finally, the hydrogel (AAc/Agar–30/70, *v*/*v*%) exhibited a thermal degradation profile resulting from the combination of both polymers. The hydrogel presented an improvement in thermal resistance compared to agar; it exhibited three well-defined decomposition curves, the first at 273 °C, which matches the degradation of agarose overlapped with the decarboxylation step for PAAc. The degradation process observed at 413 °C belongs to the degradation of the PAAc backbone; meanwhile, the degradation temperature of 865 °C belongs to the agaropectin present on the hydrogel. This analysis confirms the formation of the new polymeric system with good thermal resistance.

#### 3.2.3. Mechanical Test

**Figure 5:**
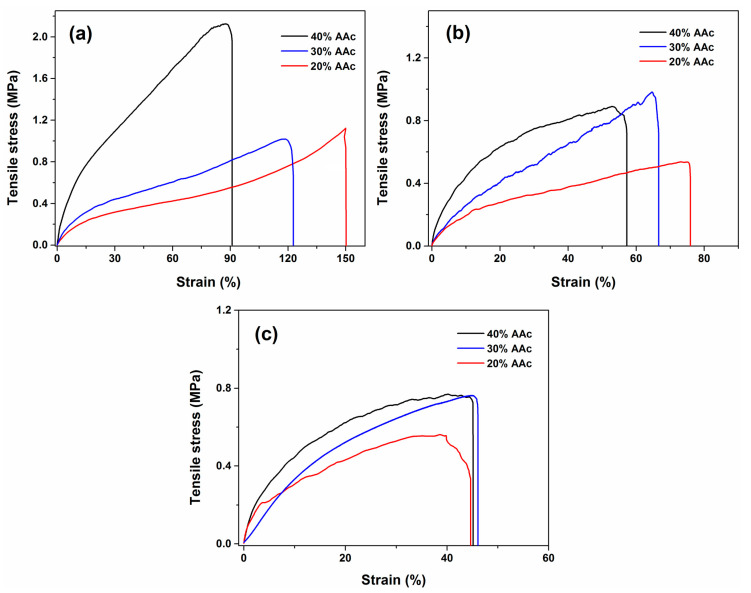
Mechanical properties of poly(Agar AAc) samples at a proportion of AAc/Agar at 20/80, 30/70, 40/60 (*v*/*v*%) irradiated with: (**a**) 15 kGy, (**b**) 20 kGy, and (**c**) 25 kGy. Representative curves of each sample were obtained from measurements carried out in triplicate.

The mechanical tests showed that the applied radiation dose and amount of AAc notably affect the breaking strength and elasticity of the hydrogel. Figure 5 presents the correlation of the tensile stress of the copolymers. Figure 5a corresponds to the samples irradiated at 15 kGy; the figure shows that the highest tensile stress (2.1 MPa) was for the sample with 40% of monomer in volume, this is because the acrylic promotes the cross-linking of the hydrogel through its vinyl group and therefore, at higher monomer molecules more covalent bonds are formed, making the polymer more rigid; the strain presented in that figure demonstrates that there is an inverse correlation between the strain percentage and the amount of acrylic acid, since the hydrogels with a lower content of the AAc presented a higher strain percentage (149%) due to a minor cross-linking degree. The samples irradiated at 20 kGy (Figure 5b) exhibited similar behavior to those exhibited for the samples from Figure 5a; the samples that contained a higher amount of AAc presented a higher rigidity compared to the samples with a minor monomer content. However, the last ones displayed more elasticity. Samples obtained at 25 kGy (Figure 5c) presented similar elongations and tensile stress among them; all the strain percentages of those samples were close to 45% (see Table 2). In this case, the effect of monomer concentration was not determinant. All the samples presented elastomeric behavior but particularly the sample with 30% and 20% of monomer at 15 and 20 kGy. The results presented for this test correspond to the average of the analyzed samples. Table 2 describes higher values in the Young’s modulus as the number of AAc increases. The samples of AAc/Agar (20/80, *v*/*v*%) show a significant increase in elongation at the break due to the amount of agar. The poly (Agar-*co*-AAc) hydrogel (30/70, *v*/*v*%) concentration presents intermediate values between hydrogels subjected to 40% and 20% of AAc and either the Young’s Modulus or the tensile strength. On the other hand, the dose parameter significantly affects the mechanical behavior; in general, the cross-linking extent, toughness, tensile strength, and resistance improve simultaneously and the elongation diminishes [33].

#### 3.2.4. Swelling Studies

**Figure 6:**
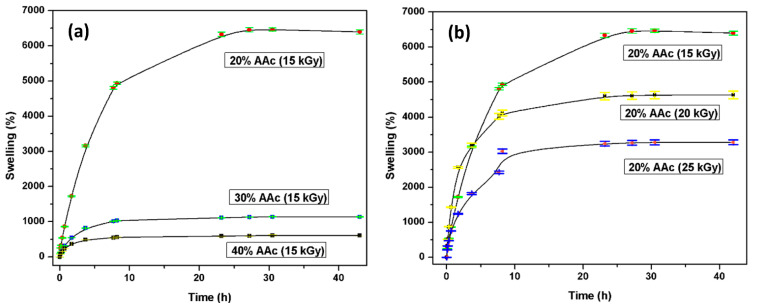
Hydrogels swelling profiles based on (**a**) monomer concentration and (**b**) applied doses.

As the polymer absorbs more solvent, the network is progressively expanded [34]. The water absorption rate directly depends on different parameters, such as the AAc concentration AAc/Agar (20/80, 30/70, and 40/60, *v*/*v*%) and the applied radiation dose (15, 20, and 25 kGy). Figure 6a illustrates the swelling test performed. Many hydroxyl groups present in the hydrogel are responsible for interacting and forming bonds with the water.

Compared with the hydrophilic groups present in AAc, it promptly forms hydrogen bonds with the surrounding environment. Therefore, when the hydrogel is exposed to high radiation doses, the degree of cross-linking between chains increases, which is inversely proportional to the water absorption capacity. As the radiation dose increases, more connection points are created in the structure, resulting in a compact system. According to Figure 6b, the sample’s water absorption rate irradiated with 15 kGy is close to 6500%, while the poly(Agar-*co*-AAc) structure, irradiated with 25 kGy, absorbs 3000% of water. Hydrogels’ high water uptake capacity would benefit injured or burned skin since these kinds of injuries are known for losing plenty of liquids.

#### 3.2.5. Critical pH

The primary goal of these studies was to evaluate the effects of different monomeric mixtures on the physicochemical behavior of the system in simulated physiological fluids such as pH. The hydrogels presented a great uptake capacity of PBS because of ions in the buffer solutions, and hydrogels kept their strength and structure. Samples performed some assays at monomer concentrations of AAc/Agar (20/80, 30/70, and 40/60; *v*/*v*%) and applied doses (15, 20, and 25 kGy). The swelling degree increased proportionally to the agar content in the copolymers. This is because hydrogels with high agar content show more hydrophilic groups readily to form bonds.

**Figure 7:**
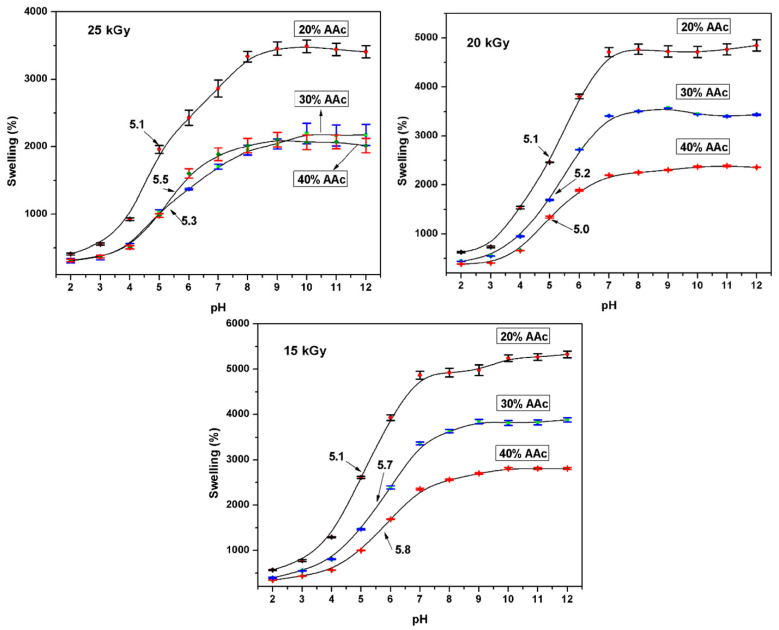
Determination of pH critical point at different parameters. Representative curves of each sample were obtained from measurements carried out in triplicate.

Hydrogels swelled fairly slowly in buffer solution and reached the equilibrium between 12 and 24 h depending on some parameters. Figure 7 showed that the critical pH point for poly(Agar-*co*-AAc) was 5.4, and it exhibits slight displacements based on acidic moiety content. At pH values above 5.4, the –COOH groups’ ionization leads to electrostatic repulsions among PAAc chains, making them adopt an expanded conformation. The pK_a_ value of poly(acrylic acid) is between 4.5 and 5.0, and PAAc hydrogels swell significantly at the physiological pH of 7.4 due to ionization of the anionic carboxylic acid groups [35]. In contrast, at pH values below 5.4, carboxylic groups of AAc are not ionized (collapsed state); consequently, the intermolecular repulsions are at a minimum.

Blank reported that the skin pH values between 4.2–5.6 [36], while Zlotogorski emphasized that forehead pH is 4.0–5.6, and for the cheek, it is 4.2–6.0 [37]. Therefore, the hydrogel presents a pH close to the skin, which could help its biocompatibility with the human tissue.

#### 3.2.6. Load and Release of Ciprofloxacin

**Figure 8:**
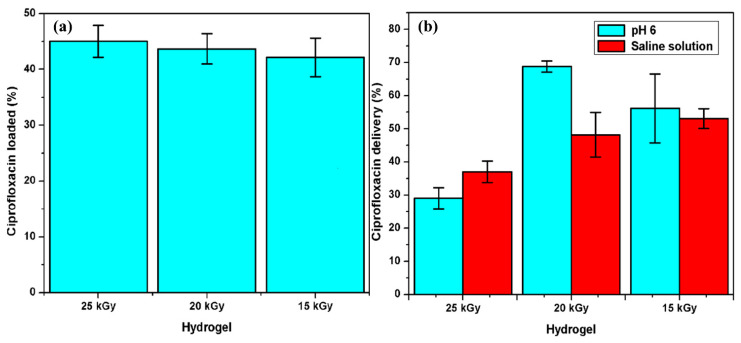
Ciprofloxacin studies: (**a**) Drug–loaded, (**b**) Drug released. All samples contain AAc/Agar (30/70, *v*/*v*%) concentration.

Hydrogels (AAc/Agar-30/70 *v*/*v*%) were immersed in 0.012 mg/mL ciprofloxacin solutions. The samples presented a significant drug adsorption capacity. It was noticed that all the hydrogels presented a good affinity to the drug; the loading efficiency increased as a function of the applied dose. This point of view could be explained by the cross-linking degree since, at higher doses, a greater number of covalent bonds can be formed; that means that the closeness between hydrophilic groups helps to retain the drug (Figure 8a). Since the number of carboxylic groups present was almost the same for three cases, the acrylic acid was 30% of the volume for all samples. Besides, the ciprofloxacin has a positive charge (due to the protonation of the amine group) [38], which favors the intermolecular interactions between the carboxyl group and the drug, resulting in a loading efficiency of about 40–45%.

Release studies were performed in a PBS of pH 6 and saline solutions (pH 6.3) at 37 °C to simulate the physiological conditions (Figure 8b). The hydrogels obtained by applying 15 and 20 kGy released more drugs than those obtained at 25 kGy; they released approximately 70% and 60% of the whole drug previously loaded in the buffer solution, respectively. However, when the hydrogels obtained at 15 and 20 kGy were tested in the saline solution, they released 50% of the drug or less after 40 h of study. This is because the ionic force of the saline solution is lower than that presented for PBS, and the polymer swells less. The sample that loaded more drug was the sample obtained at 25 kGy; however, the intermolecular interactions between both species were strong enough not to diffuse the drug to the solution but to remain in the copolymer structure.

For drug delivery systems, the less rigid but more relaxed polymer matrix structure may promote drug diffusion; meanwhile, a highly cross-linked hydrogel would slow down the diffusion of the hydrogel [19,39]. Therefore, Figure 8 depicts the limited delivery of drug as irradiation dose increases, thus following that criteria.

#### 3.2.7. Load of Silver NPs

**Figure 9:**
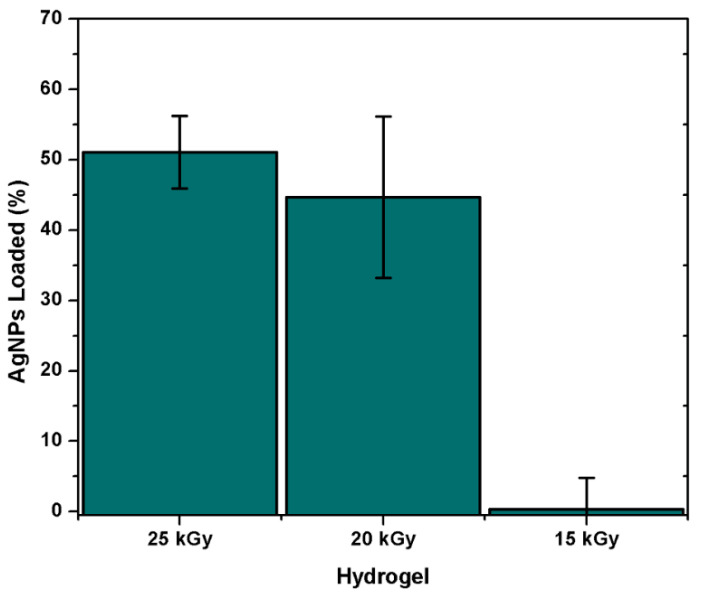
Amount of AgNPs-loaded on the polymeric matrix of the hydrogel. All samples contain AAc/Agar (30/70, *v*/*v*%) concentration.

Poly(Agar-*co*-AAc) hydrogels (30/70, *v*/*v*%) were immersed in AgNPs suspensions of 0.0784 mg/mL. The absorbance of the suspension was constantly monitored to determine the equilibrium time of absorption. The groups that present the ability to host the metallic nanoparticles are the hydroxyl and carbonyl groups present in the hydrogel, which interact with AgNPs through electrostatic interaction. Figure 9 showed an important dependence based on radiation dose. Samples irradiated at 15 kGy did not present affinity for AgNPs even after 45 h of immersion. This sample just absorbed the solution, but the solution’s absorbance did not display a notable decrease; this means that the AgNPs need a more compact structure to be retained in it. On the other side, the hydrogel synthesized with 20 kGy presented a remarkable load capacity; because of its greater cross-linking extent, this capacity was more prominent for the hydrogel obtained at 25 kGy; this sample presented the highest load efficiency, which was close to 50%. This sample displayed the highest load ability since it had a more compact structure, and the hydrophilic groups easily encircled the metallic nanoparticle due to the proximity among them.

### 3.3. Antimicrobial Activity

**Figure 10:**
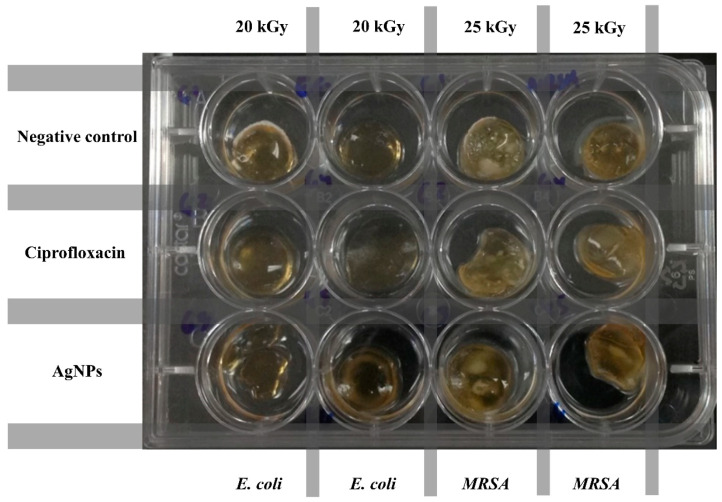
Antimicrobial assay of different hydrogels challenged against *E. coli* and *MRSA*.

The antimicrobial assay of hydrogels was carried out in a solution infused with *E. coli* and *S. aureus* (Figure 10). A hydrogel without antibiotics was used as a negative control. Ciprofloxacin was used as a drug model since it has excellent antibacterial activity against a wide spectrum of Gram-positive and Gram-negative microorganisms [40]. Ciprofloxacin has similar activity against all Staphylococci [41]. Meanwhile, AgNPs are capable of attacking and eliminating some Gram-negative (*E. coli*, *Pseudomonas aeruginosa* and *Salmonella enterica*) and Gram-positive microorganisms (*S. aureus* and *Bacillus subtilis*) [42].

**Figure 11:**
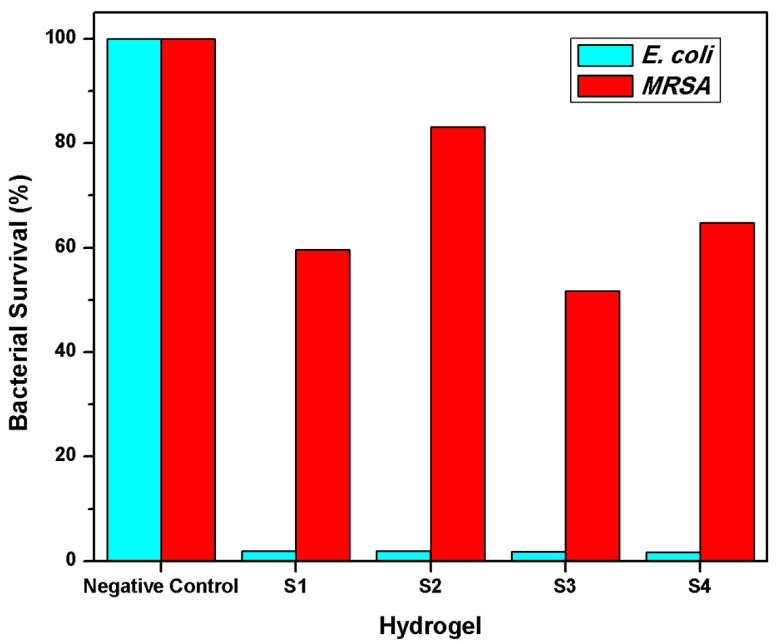
Antimicrobial effect against *E. coli* and *MRSA*. All samples contain AAc/Agar (30/70, *v*/*v*%) concentration. S1 and S2 were loaded with ciprofloxacin. S3 and S4 were loaded with AgNPs. S1 and S3 were irradiated at 25 kGy, and S2 and S4 were irradiated at 20 kGy.

Figure 11 shows the negative control, which showed 100% of bacterial survival since this sample did not contain an antimicrobial agent. The S1 and S2 are ciprofloxacin–loaded-hydrogels AAc/Agar (30/70, *v*/*v*%), irradiated at 25 and 20 kGy. On the other side, the S3 and S4 are samples loaded with AgNPs (AAc/Agar: 30/70 *v*/*v*%) at different irradiation doses. All samples displayed an excellent biocidal activity against *E. coli* since they reduced about 98% of the count on both ciprofloxacin and AgNPs loaded hydrogels. However, the efficacy when tested against *S. aureus* was notably lower than exhibited for *E. coli.* The mechanism of ciprofloxacin and other quinolones involves inhibiting bacterial DNA gyrase [43], thus reducing bacterial activity. The samples S1 and S3 exhibited a better performance in eradicating the S. aureus than the S2 and S4 samples independently of the biocidal agent; this is because S1 and S3 had a minor cross-linking extent and, therefore, release more drug and were more active against the bacteria. On the other side, the samples loaded with silver nanoparticles exhibited a higher bacterial killing percentage compared to hydrogels loaded with ciprofloxacin obtained at the same irradiation doses, respectively. In summary, the samples exhibited better antimicrobial activity against *E. coli* than *MRSA*, independently of the amount of loaded drug and biocidal agent. The lower biocidal efficacy observed for *MRSA* is because Gram-positive bacteria have a thicker cell wall, which makes them less susceptible to ciprofloxacin.

## 4. Conclusions

Highly cross-linked hydrogels were easily obtained by way of the gamma-radiation technique by reacting to cheap compounds, such as acrylic acid and agar, where acrylic acid acted as the cross-linker agent activated by the ionizing radiation. The hydrogels obtained presented a well-defined 3D shape that was unaltered by absorbing a significant amount of water. They exhibited excellent mechanical properties and elastomeric behavior; the Young’s modulus depended on the cross-linking degree, which was improved by applying high radiation doses. The enhanced cross-linking extents were obtained thanks to the high reactivity of AAc and its double bond. At high contents of AAc, more rigid structures were obtained. All the poly(Agar-*co*-AAc) samples exhibited excellent mechanical properties. However, those obtained at 15 kGy and 20% AAc exhibited the highest water uptake capacity, absorbing six thousand times their weight; this capacity was negatively affected by the content of acrylic acid and the dose, since more rigid structures were obtained with the increase of those factors. Hydrogels displayed a critical pH close to 5.3 due to the PAAc in PBS at room temperature. The loading efficiency was notably affected by the cross-linking extent of hydrogels. The samples with higher load capacity were obtained at 15 and 20 kGy; these were the hydrogels with better activity against *E. coli* and *S. aureus* than that obtained at 25 kGy regardless of the loaded biocidal agent. Poly(Agar-*co*-AAc) hydrogels have potential biomedical applications for wound healing or skin burns due to all the properties they exhibited; in addition, they presented good adhesivity to the skin, which helps the hydrogel to not move once placed over it. Our work strategy will open up a new platform to overcome the limitations of conventional hydrogels that are brittle and weak, and make progress in preparing functional materials with a well-defined 3D-shape.

## Figures and Tables

**Table 1:** Concentration and doses applied for the hydrogel synthesis.

Agar/AAc (*v*/*v*%)	Radiation Dose (kGy)	Radiation Dose (kGy)	Radiation Dose (kGy)
80/20	15	20	25
70/30	15	20	25
60/40	15	20	25

**Table 2:** Mechanical properties of poly(Agar-*co*-AAc) films.

Sample (Hydrogel)	Radiation (kGy)	Young’s Modulus (MPa)	Tensile Strength (MPa)	Displacement at Break (mm)
20% AAc	25	0.28 ± 0.35	0.78 ± 0.51	45
30% AAc	25	0.37 ± 0.40	0.78 ± 0.44	47
40% AAc	25	0.37 ± 0.36	0.48 ± 0.28	45.3
20% AAc	20	0.73 ± 0.13	0.43 ± 0.21	77.5
30% AAc	20	0.49 ± 1.13	0.95 ± 0.17	67
40% AAc	20	1.64 ± 0.24	0.86 ± 0.13	58
20% AAc	15	0.95 ± 0.23	2.15 ± 0.44	149
30% AAc	15	0.99 ± 0.31	1.01 ± 0.42	122
40% AAc	15	3.25 ± 1.29	2.13 ± 0.04	91

## Data Availability

Not applicable.
